# Cytochrome P450 and P-Glycoprotein-Mediated Interactions Involving African Herbs Indicated for Common Noncommunicable Diseases

**DOI:** 10.1155/2017/2582463

**Published:** 2017-01-31

**Authors:** Gregory Ondieki, Makafui Nyagblordzro, Siambi Kikete, Rongjia Liang, Lili Wang, Xin He

**Affiliations:** ^1^School of Chinese Materia Medica, Tianjin University of Traditional Chinese Medicine, Nankai District, Tianjin 300193, China; ^2^Tianjin State Key Laboratory of Modern Chinese Medicine, Tianjin 300193, China

## Abstract

Herbal remedies are regularly used to complement conventional therapies in the treatment of various illnesses in Africa. This may be because they are relatively cheap and easily accessible and are believed by many to be safe, cause fewer side effects, and are less likely to cause dependency. On the contrary, many herbs have been shown to alter the pharmacokinetics of coadministered allopathic medicines and can either synergize or antagonize therapeutic effects as well as altering the toxicity profiles of these drugs. Current disease burden data point towards epidemiological transitions characterised by increasing urbanization and changing lifestyles, risk factors for chronic diseases like hypertension, diabetes, and cancer which often present as multimorbidities. As a result, we highlight African herb-drug interactions (HDIs) modulated via cytochrome P450 enzyme family (CYP) and P-glycoprotein (P-gp) and the consequences thereof in relation to antihypertensive, antidiabetic, and anticancer drugs. CYPs are enzymes which account for to up to 70% of drug metabolism while P-gp is an efflux pump that extrudes drug substrates out of cells. Consequently, regulation of the relative activity of both CYP and P-gp by African herbs influences the effective drug concentration at the site of action and modifies therapeutic outcomes.

## 1. Introduction

Use of traditional herbal products as an alternative and/or to complement conventional therapies (or simply CAM) continues to be an area of interest [1]. Worldwide, a considerable proportion of the population relies, at least partly, on herbs for primary healthcare especially in the developing world [2, 3]. Estimates of CAM use in some parts of Africa are at around 80% [4]. This noticeably high CAM utilization is fueled by a number of factors such as the ease of access, relative affordability, anecdotal perception of higher safety and efficacy, and natural abundance of these products as 25% of the world's higher plants, 5400 of which have medicinal value being found on the continent [5, 6].

Herbs are not used in isolation and concomitant use with conventional medicines is estimated at 20–30% in the United States [7]. Increase in the incidence of noncommunicable diseases (NCDs) in Africa has, invariably, led to a tandem surge in concurrent herb use. In a survey in Nigeria, up to 46% of diabetes mellitus patients [8], 39.1% of hypertensive patients [9], and 65% of cancer patients used or were using herbs alongside conventional drugs [10]. This poses unique challenges because (a) most herbs lack patient information, (b) available research about herbs is scanty, (c) there is poor regulation of CAM practice, and (d) there is a general lack of disclosure of herb users to their physicians [11–13].

Phytochemically, herbs are a repository of complex mixtures of bioactive compounds [14] that can potentially alter the pharmacokinetics of coadministered prescription drugs and/or herbs, especially absorption and oxidative metabolism [1, 15]. This is possible through inhibition or induction of intestinal and hepatic drug metabolizing enzymes like CYP, as well as efflux and transport proteins which have been touted as the main mechanism for HDI [11, 16]. HDI occurs when a herbal preparation interferes with the metabolism of a coadministered conventional drug(s) by interacting with the enzyme for which it is a substrate hence causing an apparent alteration in the plasma concentration of the affected drug hence affecting its normal pharmacokinetic profile [17, 18]. The interaction is of more importance especially for drugs with narrow therapeutic window as the consequences are life-threatening [19].

By herbs modulating CYP activity, pronounced pharmacokinetic changes in a concomitantly given drug may be observed [20].

African herbs are not covered in the text but are commonly used in management of NCDs; some of their important phytochemical composition and pharmacokinetic effects on CYP and/or P-gp have been summarized in Table 1. Similarly, some selected compounds known to affect the activities of CYP and P-gp are shown in Figure 2. Invariably, use of herbs as remedy for various diseases and conditions will most likely depend on geographical location and folklore use among the populace. Therefore, in this review the classification of herbs as discussed here is based on their most common use.

## 2. Prevalence and Comorbidity of Diabetes, Hypertension, and Cancer in Africa

Dynamics of disease patterns have changed in favour of noncommunicable diseases (NCDs) on the continent [21, 22]. To a lesser extent, prevalence of NCDs appears to be influenced by genetics [23]. For instance, populations of African ancestry have a disproportionately higher burden of cancer and hypertension [24, 25].

At the beginning of the millennium, 37% of deaths in South Africa were attributed to NCDs with diabetes mellitus (DM), cancer, and cardiovascular diseases taking the lead [26]. In 2011, 30% of the 9.5 million deaths in Africa and 25.8% of the 675.4 million disability-adjusted life years recorded in Sub-Saharan Africa were NCD linked [27, 28].

About 7.7% of the world population will be diabetic by 2030 with the incidence rates in developing countries increasing by 69% as compared to 20% for their developed counterparts mainly due to increase in risk factor exposure [29]. Among NCDs, hypertension is the leading cause of debilitations and disabilities in Africa prompting the African Union to declare it the greatest health challenge after HIV/AIDS [30, 31]. With this current trend, by 2025 there will be 126 million hypertension and a predicted 49.7 million DM patients on the continent by 2030 [32]. The crude prevalence of cancer in Africa is estimated at 0.3% [33] and those reported to be highly prevalent in Africa include ovarian, hepatobacillar, Kaposi's sarcoma, breast, and prostate cancers [34].

NCDs show comorbid tendencies. A survey in the US showed that up to 68.7% of cancer patients had other chronic condition(s) including DM and hypertension [35]. A cohort study by Gerber et al. revealed that there was 71% comorbidity rate for hypertension, cancer, and diabetes in elderly South Africans [36]. In another survey among primary healthcare attendants in Western Cape, South Africa, 23% of hypertension patients had DM and 40% of DM patients had hypertension [37].

## 3. Role of CYP450 and P-Glycoprotein in Drug Metabolism

### 3.1. CYP450

**Figure 1:**
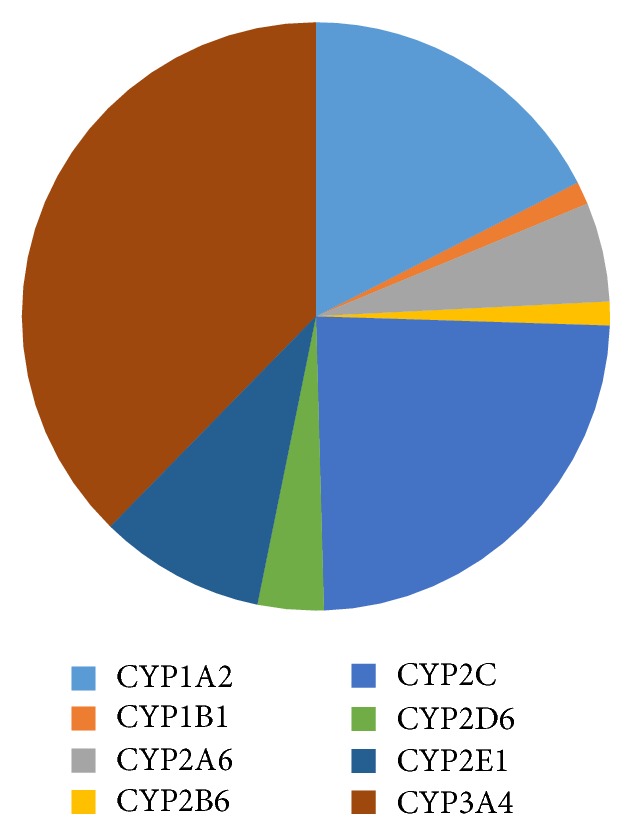
Relative abundances of important hepatic CYPs, adopted from Rendic and Di Carlo [40].

**Figure 2:**
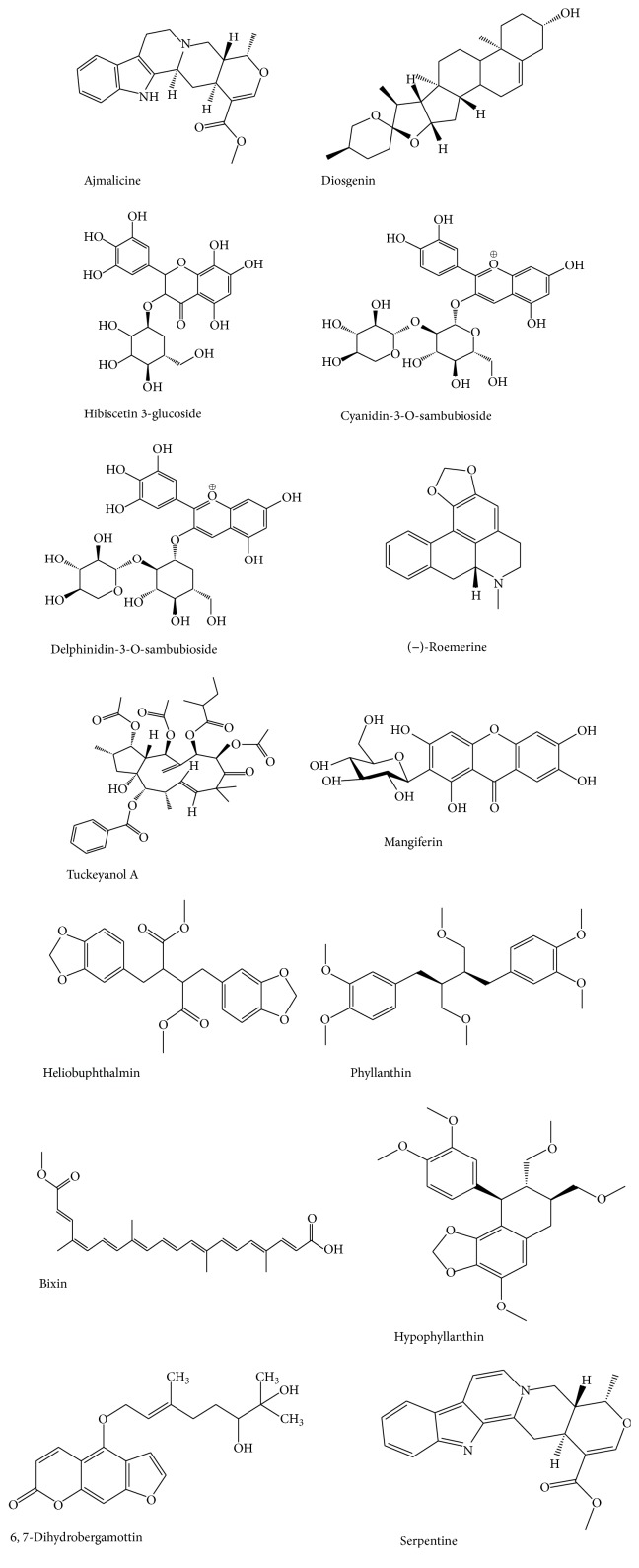
Selected phytochemicals extracted from African herbs that modulate CYP or P-gp.

CYP450 refers to a large family of iron-containing enzymes involved in phase I metabolism of exogenous or endogenous chemical entities where they act by introducing and/or exposing a polar moiety via oxidation, hydrolysis, or reduction [38]. Their classification is based on families. Those belonging to families 1, 2, and 3 are the principal xenobiotic metabolizers (Figure 1). The others are involved in biotransformation and elimination of various endogenous biomolecules such as fatty acids and hormones [39].

These oxidative enzymes account for up to 80% of phase I metabolism of therapeutic drugs in use [41]. CYP3A is the most dominant CYP and is the culprit in most drug-drug interactions [42]. Although present in many organs, the vast majority of the oxidative CYP450 enzyme systems are domiciled in the liver [43], with other organs, for instance, the small intestine being mostly dominated by CYP3A, in particular, CYP3A4 and CYP3A5 [44]. The review will focus on all the important CYPs and consequences of their inhibition or induction.

### 3.2. P-Glycoprotein

P-Glycoprotein is a transmembrane ATP-dependent efflux family of proteins that acts as a pump for a wide variety of chemically diverse substrates [45]. It is strategically located in various human tissues including the apical surface of intestinal epithelial cells, apical membrane of enterocytes, and renal tubules. In addition, it is expressed in tumour cells [46–48]. The human small intestines express P-gp or human multidrug resistance gene (MDR 1), multidrug resistance-associated protein 2 (MRP2), and breast cancer resistance protein (BCRP) which play a critical role in drug pharmacokinetics such as absorption, distribution, and elimination by their efflux action [49, 50]. MDR1 expressed by tumour cells affects chemotherapeutic drug concentration in these cells and in the process confers resistance to them [51]. It has been well demonstrated that P-gp is a victim of inhibition, induction, or activation by drugs just like CYP; thus it is a major cause of clinical drug-drug/herb interactions if and when they are concurrently administered [45, 52]. Inhibition of P-gp leads to enhanced bioavailability of its substrate molecules [53]. The converse is true where there is induction.

Induction of P-gp or drug metabolizing enzyme can lead to therapeutic failure and goes unnoticed especially in cancer treatment [16]. This phenomenon can, alternatively, be exploited to positively alter the pharmacological properties of some drugs [54]. P-gp expression and activity have been known to increase with age [55] and certain infectious diseases [56] and in prolonged cancer chemotherapy [51].

The effect on drug pharmacokinetics with regard to herbs encompasses all aspects of absorption, distribution, metabolism, and excretion [91] as well as genetic regulation of certain metabolic proteins [92]. Most of these aspects have been aptly covered by other authors and we will limit our scope to interactions involving P-gp and CYPs as pertains to African herbs.

## 4. African Herbs Used for Common NCDs Modulating CYP450 or P-gp Function and Their Interactions

### 4.1. Diabetes Mellitus

Diabetic condition is physiologically heralded by sustained hyperglycaemia [93] and if it is undetected and/or effectively managed, its long term complications including organ failure, blood vessels, and nerve damage are debilitating [94]. The pharmacological mainstay in diabetes management is by the use of oral antidiabetic drugs (OADs) broadly classified as biguanides, glitinides, sulfonylureas, or thiazolidinediones as well as insulin for effective hypoglycemic control [95]. It has been observed that patients on identical OAD regimens exhibit notable variations in glycaemic control, efficacy, tolerability, and adverse effects, a phenomenon attributed to differences in genes leading to varying expression of CYP and drug transporters involved in various aspects of drug metabolism and disposition of these drugs [96]. Similarly, owing to their complex phytochemical composition, when herbs are concomitantly administered with OADs or any other conventional drug(s) indicated for NCDs, modification of pharmacological effects of these drugs cannot be ruled out. It is important therefore that specific effects on metabolic enzymes and drug transporters of these useful herbs and the components isolated from them are known and documented to ensure their safe medical utilization.

#### 4.1.1. *Trigonella foenum-graecum* (Fenugreek)

*Trigonella foenum-graecum* is a herb in Leguminosae family that grows in Mediterranean areas of Africa and is used in management of diabetes and hyperlipidaemia [97, 98]. Phytochemically,* Trigonella foenum-graecum* (*T. foenum*) contains, among other compounds, tannic acid, volatile oils, fixed oils, fiber, steroidal saponins, vitamins A, B1, B2, B3, and C, flavonoids, coumarins, and amino acids [99–101].

CYP2D* m*RNA expression was depressed in rats put on a seven-day regimen of* T. foenum* as determined by a reduction in dextromethorphan metabolism [102]. In a related in vitro experiment using human liver microsomes,* T. foenum* extract at concentrations of 50–100 *μ*g/mL inhibited both CYP3A4 and CYP2D6 [103]. Another investigation using tolbutamide as a substrate showed a dose-related inhibition of gene expression coupled with a reduction in metabolic activity of CYP2C11 [104]. Concomitant administration of antidiabetic drug glibenclamide with* T. foenum* significantly increased the plasma concentration of the former in beagle dogs via an interaction whose mechanism is unknown [105]. However, diosgenin-a steroidal saponin contained in fenugreek has been shown to significantly inhibit CYP3A4 [106]. This may be one of the compounds involved and further research may reveal others involved in CYP modulation.

#### 4.1.2. *Kalanchoe crenata* (Dog's Liver)

*Kalanchoe crenata* is a herbaceous plant of the Crassulaceae family native to Madagascar and widespread in tropical Africa [107]. It is popular in parts of Cameroon where it is traditionally used to treat diabetes. Phytoconstituents of* K. crenata* include tannins, terpenoids, saponins, polysaccharides, alkaloids, and flavonoids.

An in vivo study showed that water-alcohol extract of the plant caused hypoglycaemia and an increased glucose sensitivity analogous to glibenclamide [108]. In a recent in vitro study, a methanol extract and various fractions of the herb were incubated with human hepatic microsomes or recombinant human CYP3A4 and CYP2C19 in a cocktail experiment. The extract and three fractions showed a considerable inhibition of CYP3A4 in a time-dependent manner with the extract being the most potent. Also, the crude extract and one fraction exhibited a time-dependent inhibition of CYP3A4 [109].

#### 4.1.3. *Nigella sativa* (Black Seed)

*Nigella sativa* is a herbaceous plant belonging to Ranunculaceae family.* N. sativa* is native to countries in the Mediterranean region where it is used as a spice and for treatment various conditions including diabetes and hypertension [110]. Several important compounds have been identified in the volatile oil of* N. sativa* seeds including thymoquinone, thymohydroquinone, thymol, carvacrol, nigellidine, and *α*-pinene [111]. In an in vivo study in rabbits,* N. sativa* volatile oil extract given intraperitoneally at 50 mg/kg caused a considerable reduction in blood sugar [112]. A study demonstrated that* N. sativa* seeds extract dramatically inhibits the intestinal electrogenic absorption of glucose in vitro [113]. It has been demonstrated that* N. sativa* seeds extract possesses hepatic CYP3A4 and CYP2D6 inhibition both in vitro and in vivo [114] and this may be of pharmacokinetic impact on OADs which are substrates of these enzymes.

In an in vivo study, administration of* N. sativa* for a week resulted in significant reduction of gene expression as well as metabolic activity of CYP2D [102]. A similar experiment using tolbutamide as a substrate showed that its oil extract had considerable inhibitory effects on CYP2C11 [104]. However, when* N. sativa* was coadministered with cyclosporine in an animal model, the oral bioavailability of cyclosporine was significantly reduced suggestive of the possibility that* N. sativa* causes induction of intestinal P-gp and/or CYP3A4 [115]. The ability of this herb to either induce or inhibit CYP3A4 and the potential for HDIs thereof, especially with regard to OADs, may need to be studied further.

#### 4.1.4. *Phyllanthus amarus* (Gulf Leaf Flower)

*Phyllanthus amarus* is a herb in Euphorbiaceae family. It is broadly considered as a hepatoprotective and hypoglycaemic agent by traditional practitioners as well as conventional researchers [116, 117]. It is rich in bioactive compounds including lignans, terpenoids, alkaloids, coumarins, tannins, polyphenols, and flavonoids [118]. An in vitro study showed that ethanolic extract of* P. amarus* significantly inhibited CYP3A5 and CYP3A7 [60].* P. amarus* whole plant, as well as its roots, stem, and leaf extracts, showed considerable inhibition of CYP1A2 and CYP2C9 across a range of concentrations [119]. In another study, water and ethanol extracts of* P. amarus* demonstrated a significant dose-related inhibition of human hepatic CYP1A2, CYP2D6, CYP2E1, and CYP3A4. This effect was superior to that caused by ketoconazole. Two lignans derived from* P. amarus*, hypophyllanthin and phyllanthin, showed a mechanism based inhibition (MBI) of CYP3A4 [120]. In MBI, the reactive oxidative metabolites bind covalently to CYP enzymes hence deactivating them in a more permanent manner as opposed to reversible inhibition. The inhibition effect of* P. amarus* on CYP3A4, therefore, may be more physiologically prolonged. In an in vivo study, there was a significant increase of the AUC, *C*_max_, *T*_max_, and *T*_1/2_ of midazolam when rabbits were pretreated with 500 mg/kg of* P. amarus* extract for 7 days prior to administration of midazolam, a CYP3A substrate [121]. In a related study in rats, a single dose of* P. amarus* showed significant inhibition of intestinal CYP3A. Interestingly, repeated dosing had a modest induction effect on hepatic CYP2B and CYP3A. This dual effect shows potential interaction with drugs known to be inducers or inhibitors of the affected enzymes [122].

#### 4.1.5. *Lepidium sativum* L. (Pepper Grass)

*Lepidium sativum* is a herb belonging to Brassicaceae family and is native to Morocco where traditionally its seeds are used to manage diabetes [123]. The seeds are used in diabetes management and contain free fatty acids, amino acids, tocopherols, and several phenolic compounds including quercetin [124]. In one study, 10 mg/kg/h of* L. sativum* water extract given intravenously in normal and diabetes-induced rats led to a decrease in blood glucose with a concomitant increase in urinary glucose in both groups. The extract may be exerting its hypoglycaemic effect renally by decreasing glucose reuptake [125]. In an in vitro study, ethanolic extract and seed powder of* L. sativum* when incubated with human liver microsomes showed significant inhibition of CYP2D6 and CYP3A4. The in vitro outcome was confirmed in vivo as the inhibition was corroborated in healthy human subjects. This implies the presence of a higher degree of clinical HDI interactions [126]. Its effect on CYP2D at the gene level has also been investigated and analysis of rat hepatic CYP2D* m*RNA expression after a 7-day regimen on* L. sativum* extract showed that the herb reduced the production of CYP2D and caution may be necessary when substrates of the affected CYP are used with the herb [102]. When coadministered with cyclosporine in an animal model,* L. sativum* had a statistically insignificant increase in AUC and clearance of cyclosporine. However, the elimination rate of cyclosporine was significantly decreased due to inhibition of CYP3A and modulation of intestinal P-gp [115]. Its effect on CYP2D at the gene level has also been investigated and analysis of rat hepatic CYP2D* m*RNA expression after a 7-day regimen on* L. sativum* extract showed that the herb caused a significant reduction in the production of CYP2D [102]. Therefore, its effects on CYP2D substrates can be present even where there is no concomitant administration.

### 4.2. Hypertension

The main goal of therapy in the treatment of hypertension is to lower systemic blood pressure down to an optimum range via the use of antihypertensive medications and other nonpharmacologic therapies. Owing to that, the frequently used classes of antihypertensive drugs, in particular, beta-blockers, calcium channel blockers (CCB), angiotensin converting enzyme (ACE) inhibitors, and diuretics, incidentally, are used in combination with herbal medications [127, 128]. In addition, some antihypertensive drugs are not devoid of CYP450 enzyme metabolism and P-gp interactions, and hence coincidental herd-drug interactions emanating from CYP450 substrates and the seemingly innocuous herbal medicines used in managing hypertension might trigger unknown reactions.

*Artemisia herba-alba* and* Hibiscus sabdariffa*, so far, have been researched at the level of CYP450 microsomal enzymes and its blood pressure lowering capabilities, but interactions relating to P-gp still remain elusive.

#### 4.2.1. *Artemisia herba-alba* (White Wormwood)

*A. herba-alba*, native to Northern Africa [129, 130] and in Errachidia province of Morocco, is a major herbal drug used in the management of essential hypertension [131].

Zeggwagh et al. demonstrated that the aqueous extract of white wormwood extensively ameliorated spontaneous hypertension in rats [132] leading to the identification of sesquiterpene lactones as among the most pharmacologically active compounds [133, 134]. Other bioactive phytochemicals include herbalbin, cis-chrysanthenyl acetate, flavonoids (hispidulin and cirsilineol), and monoterpenes [135, 136].

The essential oils of* A. herba-alba* were found to inhibit angiotensin converting enzyme (ACE) [137] while the sesquiterpenes showed CYP3A4 and CYP2D6 inhibitory activities when tested against erythromycin and dextromethorphan [138, 139]. The flavonoid *α*-thujone is principally metabolized in the human liver mainly by CYP2A6 followed by CYP3A4 and to a lesser extent CYP2B6. Nevertheless, its inhibitory and inductive capabilities remain unstudied [140].

#### 4.2.2. *Hibiscus sabdariffa* (Hibiscus/Roselle)

Hibiscus is native to West Africa and is believed to have originated from Sudan. Folkloric uses include food, beverage (tea), and medicine where it is believed to have antihypertensive properties with flavonoids (delphinidin-3-O-sambubioside and related compounds) being responsible for these effects [141].

Clinically,* H. sabdariffa's* potency in controlling moderate essential hypertension [142] was comparable to captopril [143]. It was also reported to increase urine outlet and mean AUC when coadministered with hydrochlorothiazide [144].

Ethanolic extracts were found to inhibit CYP1A2, CYP2A6, CYP2B6, CYP2C8, CYP2C9, CYP2C19, CYP2D6, CYP2E1, and CYP3A4 at concentrations of 306 *μ*g/mL to 1660 *μ*g/mL in vitro. [145]. On the contrary, aqueous extract of* H. sabdariffa* was shown not to have any significant inhibitory or inductive effects on CY P450 enzymes CYP1A1, CYP1A2, CYP2B1/2, CYP2E1, and CYP3A, and neither did it have any effects on the total CYP450 levels [146].

### 4.3. Cancer

Cancer cells often become simultaneously resistant to multiple drugs. The molecular basis of MDR is the overexpression of ATP-binding cassette (ABC) transporters, an MDR1/ABCB1 gene encoding P-glycoprotein which effectively extrudes hydrophobic drugs out of cancer cells, effectively precluding their activity [147, 148]. From a clinical standpoint this phenomenon correlates positively with poor therapeutic outcomes [149]. This led to the concept of chemosensitization which involves the coadministration of a P-gp inhibitor with an anticancer drug in order to enhance intracellular anticancer drug accumulation via impairing the P-gp efflux function. Early generation P-gp inhibitors induced CYP3A4-mediated drug metabolism [150] and were associated with severe toxicities. Third-generation inhibitors appear to possess acceptable toxicity profiles but are yet to find proper clinical application [151, 152] thus necessitating a possible fallback to natural products such as African herbals. Several phytochemicals were tested in vitro and found to reverse MDR but data on safety and efficacy is insufficient [153]. Since P-gp plays an important role in the intestinal absorption, distribution to the central nervous system, and biliary/urinary excretion of drugs, potential interactions may cause altered absorption and bioavailability of P-gp substrates [52, 154]. In addition, numerous African plant extracts and their phytoconstituents were shown to possess intrinsic anticancer effects both in vitro and in vivo but the majority of investigations did not demonstrate significant P-gp or CYP modulation [155–157]. Furthermore, pleiotropic or broad spectrum drug resistance is not limited to ABC transporters alone; oncogenes and tumour suppressors can also confer drug resistance in addition to their role in carcinogenesis [158, 159].

#### 4.3.1. P-gp Mediated Interactions

These herbs are characterised by limited intrinsic cytotoxicity to MDR tumours but are capable of resensitizing resistant cells to conventional cytotoxic agents via P-gp. 

*(1) Acokanthera oppositifolia (Common Poison Bush).* This plant is native to Eastern and South Africa [160] where it is traditionally used as a highly effective arrow poison [161]* A. oppositifolia* contains cardiotonic steroids, acovenoside, and ouabain.

Its extracts exert considerable dose-dependent cytotoxicity towards both sensitive CCRF-CEM and P gp overexpressing CEM/ADR5000 leukemia cells [156] as well as towards TK10 renal cancer cells, MCF-7 breast, and UACC62 melanoma cells [162]. Ouabain is known to act by inhibiting Na+/K+-ATPase leading to strong inotropic effects on the heart [163]. Cardiotonic steroids also inhibit the efflux function of P-glycoprotein and overcome MDR of tumour cells [164]. Saeed and group found that at lethal concentrations acovenoside A and ouabain did not reveal any statistically significant associations between cellular responsiveness to the two cardiotonic glycosides and ABCB1 expression and function. On the contrary, Brouillard et al. earlier reported that ouabain treatment led to the induction of MDR1 expression [165] leading the latter authors to hypothesize that this likely occurred at sublethal doses. Furthermore, Saeed and colleagues further documented an association between high ABCC1 expression and resistance to the two glycosides could not be established in the NCI cell line, and they concluded that ABC transporter had a limited role in the unresponsiveness of NCI cell line to acovenoside A and ouabain [156]. As such these contradictory findings require further in-depth investigation in order to ascertain actual interaction of these steroids with ABC transporters and the possible clinical implications thereafter. 

*(2) Annona senegalensis (Wild Custard Apple).* It is native to tropical east and northeast, west and west-central, and southern Africa, as well as southern subtropical Africa. Stem bark and leaves are used for the treatment of skin cancer and leukemia [166].

In vitro leaf extracts of* A. senegalensis* show limited intrinsic cytotoxicity against various cell lines [167, 168]. Interestingly, its aporphine alkaloid, (–)-roemerine, increased the toxicity of vinblastine to MDR oral epidermoid carcinoma KB-V1 cells, possibly through the inhibition of substrate binding in P-gp, therefore decreasing cellular efflux of the anticancer drug [168]. Therefore further studies are required to validate the possible benefits of coadministration of these related compounds in chemotherapy. 

*(3) Euphorbia tuckeyana (Tortolho).  E. tuckeyana* is endemic in Cape Verde. The macrocyclic diterpenes of the jatrophane type, tuckeyanols A and B, and euphotuckeyanol, isolated from aerial parts of* E. tuckeyana*, were tested for P-gp modulating properties on human MDR1 gene-transfected and parental L5178 mouse lymphoma cell lines. All compounds were found to highly increase Rhodamine-123 retention in the cells by inhibiting the efflux pump activity mediated by P-gp, resulting in a reversal of MDR. This activity was concentration dependent and found to be more potent when compared with the positive control verapamil. These compounds also exhibited moderate antiproliferative activity on the two cell lines [169]. Their strong P-gp modulation on both human and murine MDR1 cell lines makes them good candidates for further clinical investigations.

*(4) Mangifera indica (Mango).* Originally from India, Mango is naturalised in tropical Africa. Folkloric uses include the treatment of fever, jaundice and liver disorders [170], and arthritis and type II diabetes [171]. Mangiferin, a xanthone glycoside, is a major bioactive constituent that has strong antioxidant, antilipid peroxidation, immunomodulation, cardiotonic, hypotensive, wound healing, and antidegenerative and antidiabetic activities [172].

When MCF-7, a model cell line for human mammary carcinoma, was incubated with doxorubicin for 10 days, it showed decreased sensitivity to the drug. The addition of mangiferin at concentration 50 *μ*M restored sensitivity to doxorubicin. Treatment of the cells with verapamil (positive control for P-gp and MRP inhibitor) and nelfinavir (positive control for BCRP) also achieved similar results. At this concentration, mangiferin showed a significant reduction of P-gp mRNA expressions but did not influence the mRNA expressions of MRP1 and BCRP suggesting that modulation of P-gp alone may be sufficient for mangiferin mediated tumour sensitization [173].

In vitro human hepatocytes treated with subcytotoxic concentrations of mangiferin showed concentration-dependent inhibition of five P 450 enzymes, that is, CYP1A2, CYP2A6, CYP2C6, CYP2D6, and CYP3A4 [174]. This may result in increased bioavailability and possibly toxicity of coadministered anticancers like vinca alkaloids and taxane alkaloids which are CYP3A4 substrates.

*(5) Pycnanthus angolensis (African Nutmeg).* African nutmeg is widely spread in tropical Africa. In traditional African medicine the sap was used to control bleeding. The bark was used as a poison antidote and a treatment for leprosy, anaemia, infertility, gonorrhea, and malaria.

The lignans (−)-dihydroguaiaretic acid and heliobuphthalmin were isolated from the chloroform extract of* P. angolensis* and tested against cancer cells expressing classical MDR phenotype. Heliobuphthalmin showed significant antiproliferative activities against the subline EPG85-257RDB that overexpresses MDR1/P-gp which was 8-fold more sensitive than the parental drug-sensitive cells. On the other hand (−)-dihydroguaiaretic acid showed a moderate activity against multidrug-resistant EPG85-257RNOV cells [175]. In addition, seven lignans either isolated from* P. angolensis* or obtained by derivatization were strong inducers of apoptosis of human hepatoma HuH-7 cells [176]. Therefore, heliobuphthalmin may be a promising candidate as a chemosensitizing agent for consideration in drug development.

*(6) Sutherlandia frutescens (Cancer Bush).* This plant is endemic to southern Africa and referred to as “cancer bush” because of the widespread use by the Khoisan and Zulu in treatment of internal cancers. Ethnomedically, it was also employed in treatment of influenza, wounds, pains, aches, and skin disorders [177]. Triterpenoid and flavonol glycosides (kaempferol and quercetin glycosides) have been isolated from* S. frutescens* [178, 179].

Extracts of* S. frutescens* have shown antiproliferation and apoptotic effect in breast cancer cells and cervical cancer cells [180–182]. Its actual mechanism of action remains largely unknown but in vitro* Sutherlandia* represses Hedgehog-signaling pathway in murine prostate cancer cells [183] as well as key molecules in the PI-3 K pathway in colon cancer cells [184].

*Sutherlandia* extract exerted inhibitory activity on the transport activities of intestinal P-gp with an IC_50_ 324.8 *μ*g/mL but did not exert any inhibitory activity on the activity of BCRP [185] indicating that at high doses the extract may cause an increase in bioavailability of susceptible drugs. Interestingly, kaempferol markedly increased the sensitivity of the multidrug-resistant human cervical carcinoma KB-V1 cells to vinblastine and paclitaxel dose-dependently and also decreased the relative resistance of these anticancer drugs in KB-V1 cells [186].

In healthy male subjects, administration of* S. frutescens* tablets and then a single dose of atazanavir (a substrate of both intestinal P-gp and CYP3A4) caused a significant reduction in the bioavailability of atazanavir which may be attributable to a decrease in absorption and/or enhanced metabolism of atazanavir [187]. Since this drug is a substrate for both, we cannot conclude whether reduced bioavailability is attributable to the transporter or the enzyme. However, chronic dosing of* Sutherlandia* reduced the plasma levels of a single dose of nevirapine in rats, which correlated with an increase in the expression of CYP3A4 [188]. In vitro,* Sutherlandia* showed a concentration-dependent inhibition of CYP1A2, CYP2A6, CYP2B6, CYP2C8, CYP2C9, CYP2C19, and CYP3A4/5 [185]. Therefore, we expect that coadministration of this herb in sufficient doses and anticancers, the majority of which are CYP3A4, may result in increased bioavailability and possibly toxicity.

#### 4.3.2. Non-P-gp Mediated Interactions

These herbs are characterised by significant intrinsic cytotoxicity to cancer cells mediated via a variety of molecular mechanisms exclusive of P-gp. 

*(1) Catharanthus roseus (Madagascar Periwinkle).  Catharanthus roseus* is native to Madagascar and is the source of cytotoxic vinca alkaloids like vincristine and vinblastine which are the second most used class of anticancers in conventional medicine [189]. Their cytotoxicity was reported to be impaired in MDR [190, 191]. As a result several in vitro studies investigated the resensitization of MDR cells to vinblastine and are discussed above [168, 192, 193]. Although the authors reported beneficial findings, one needs to be cautious in extrapolating these to suggest resounding clinical advantages. Nevertheless, should future findings prove that benefits of chemosensitization with African herbals outweigh risks, then negative dose adjustment of anticancers may be necessary.

Repeated administration of vinblastine, which is standard practice in chemotherapy, increased both activity and gene expression of CYP3A4 in humans and mice [194]. As such the clearance of coadministered substrates of this enzyme may be increased thus compromising their plasma concentration possibly resulting in suboptimal clinical outcomes.

*(2) Hypoxis hemerocallidea (African Potato).* This herb belongs to the family Hypoxidaceae. It is indigenous to and grows widely in the southern Africa savanna grasslands [195]. The rootstock was widely used traditionally particularly in Zulu society for various chronic conditions, infections among other indications [196]. Contemporary uses in ACM today include the management of HIV/AIDS, arthritis, myalgic encephalomyelitis (ME), hypertension, asthma, diabetes mellitus, cancer, arthritis, psoriasis, tuberculosis, and epilepsy [197–199].

Hypoxoside is the major diglucoside isolated from the corms of this plant. Intragastrically, it is deconjugated by *β*-glucosidase to form the cytotoxic and lipophilic aglycone, rooperol, which is a potent inhibitor of leukotriene synthesis in polymorphonuclear leucocytes at concentrations of 1 *μ*M or less [200]. Substantial cytotoxicity against Murine BL6 and human UCT-Mel 1 melanoma cell lines was reported at 50 *μ*g/mL in presence of *β*-glucosidase. [201]. Chloroform extracts of* H. hemerocallidea* and* H. sobolifera* were also cytotoxic against MCF-7, HeLa, and HT-29 cancer cells [202].

In vitro, water extracts of* Hypoxis hemerocallidea* showed no inhibitory activity against P-gp and BCRP at concentrations of up to 500 *μ*g/mL. However Fasinu et al. observed a concentration-dependent inhibition of CYP1A2, CYP2A6, CYP2B6, CYP2C8, CYP2C9, and CYP3A4/5 with the extract at concentrations of 100 *μ*g/mL [203]. The herb is widely used in Africa as adjunct treatment in many chronic ailments and as such can alter pharmacokinetics of drugs such as calcium channel blockers and chemotherapeutic agents stated above by increasing plasma concentrations to above safety margins. Fasinu et al. performed single dose experiments which may not be used to accurately predict clinical outcomes because chronic use of the herb is the norm and may thus predispose patients to even graver potential CYP interactions.

## 5. Clinical Consequences of Modulation of Cytochrome P450 and P-Glycoprotein

### 5.1. Cytochrome P450

*T. foenum, K. crenata, N. sativa, P. amarus, L. sativum, A. herba-alba, H. sabdariffa, M. indica, S. frutescens*, and* H. hemerocallidea* have shown potential to inhibit CYP3A4. In fact, most of the herbs under consideration have an effect on this particular CYP. This is as expected because, as indicated earlier, CYP3A4 is the most critical enzyme in metabolism of drugs commonly used in management of NCDs. Its inhibition therefore means a higher possibility of HDIs and consequent precipitation of adverse drug events by these drugs.

Sulphonylureas like glibenclamide are mostly metabolized by CYP3A4 [106, 204] and so are thiazolidinediones including pioglitazone and troglitazone [205]. As a precaution, the doses of the respective OADs for patients taking these herbs need to be adjusted downwards in order to achieve the desired glycaemic parameters.

Repeated administration of* A. herba-alba* or* H. sabdariffa* incorporated herbal remedies with CYP3A4 substrates like calcium channel blockers, losartan, and nateglinide [206] could trigger untoward effects. Thus, coadministration with amlodipine may decrease the metabolism of amlodipine, where a higher plasma concentration and a potential side effect such as hypotension are conceivable. Correspondingly, nateglinide is partly metabolized (30%) by CYP3A4 [207] and could be affected by inhibitors of CYP3A4. Furthermore, the majority of currently prescribed antineoplastic agents are metabolized by CYP3A4, for instance, docetaxel, erlotinib, imatinib, irinotecan, paclitaxel, and vinca alkaloids [208].

Some herbs may have an induction/inhibition effect on CYP. A good example is* N. sativa*. When* N. sativa* was coadministered with cyclosporine in an animal model, the oral bioavailability of cyclosporine was significantly reduced suggestive of the possibility that* N. sativa* causes induction of intestinal P-gp and/or CYP3A4 [115]. The ability of* N. sativa* to either induce or inhibit CYP3A4 and the potential for HDIs thereof, especially with regard to OADs, may need to be studied further.

CYP2C8 and CYP2C9 are principal metabolizers of several drugs used for NCDs especially OADs.* T. foenum, L. sativum, P. amarus, H. sabdariffa, S. frutescens*, and* H. hemerocallidea* among others inhibit CYP2C8 and/2C9. This is a potential interaction point especially in diabetes patients on OADs because glitazones like rosiglitazone are largely metabolized by CYP2C8 and to a lesser extent by CYP2C9 [209]. This means that diabetes patients using glitazones and the candidate herbs concurrently may be at risk of hypoglycaemia as well as side effects associated with glitazones.

Likewise, CYP2C19 is involved in metabolism of the tolbutamide (a sulphonylurea) as well as antihypertensives like propranolol [210]. It is inhibited by, among others,* K*.* crenata* and* S. frutescens*. Hence, in diabetic patients with comorbid HTN on a combination of these medications and the stated herbs, there may be a risk of hypoglycaemia and inadequate reduction in blood pressure.

CYP2D6 is important in metabolism of some antihypertensives such as propranolol, alprenolol, and verapamil [211].* T. foenum, L. sativum, A. herba-alba*, and* H. sabdariffa* have all been shown to be inhibitors of CYP2D6 and this is a potential source of HDIs. As a result, they possess the likelihood of inducing synergism or an increased bioavailability of the said drugs due to reasons as have been noted. Beta-blockers like propranolol have a narrow therapeutic window and as such caution is necessary and dose adjustment may be of help. CYP2D6 inhibition may however be of little consequence in patients on OADs as it plays a minor role in metabolism of these types of drugs.

### 5.2. P-Glycoprotein

The influence of P-gp on cancer management is two-pronged. At the intestinal lumen P-gp extrudes susceptible drugs back into the lumen. As a consequence their bioavailability may be diminished.* S. frutescens* is one herb that has been tested against intestinal P-gp and shown to inhibit it if sufficient concentrations are achieved albeit under in vitro conditions. The expected clinical outcome of this interaction would be increased bioavailability and possibly toxicity in view of the fact that most anticancers have a narrow therapeutic window [212]. Nonetheless, should the intrinsic cytotoxicity of such herbs be significant, then they could be utilized as adjunct therapies with conventional anticancers whose dose may be adjusted downwards. However extensive studies are required to validate this proposition.

At the tumour cell membrane chemosensitization can be achieved by inhibiting P-gp. Several African herbals were confirmed to inhibit this transporter thus improving sensitivity of cancer cells to a myriad of conventional anticancers like paclitaxel, doxorubicin, and vinca alkaloids which also happen to be the second most used anticancers and are derived from the African plant* Catharanthus roseus*. Compounds like tuckeyanols and heliobuphthalmin show strong reversal of resistance in cells expressing MDR1/P gp genes and are therefore good candidates for further drug development. Clinically, they could be coadministered with P-gp substrates, where P-gp inhibition would result in dug accumulation to cytotoxic concentrations and ultimately improve therapeutic outcomes.

Not all herbs were reported to inhibit MDR1 expression. Brouillard et al. reported that ouabain, a compound in* Acokanthera oppositifolia*, can induce MDR expression in vitro. The clinical implications of this would be catastrophic if the herb was coadministered with anticancer drugs. On the contrary, Saeed et al. reported that there was no correlation between cellular responses to ouabain by cancer cells and ABCB1 expression and function. Interestingly several authors reported that there was a dose-dependent cytotoxicity of extracts of* A. oppositifolia* to both sensitive and MDR resistant cells. These contradictory findings point to the need for more extensive research in this area.

In addition, individual variations in clinical outcomes may be influenced by P-gp genetic polymorphisms. For instance, the association of wild-type ABCB1 (P-gp) CGC haplotype with a slower clearance may suggest that, in individuals who possess this P-gp genetic polymorph, P-gp-mediated transport of anticancers is less significant compared to those who exhibit other P-gp polymorphs. These individuals may therefore be at a lower risk of toxicity mediated through P-gp inhibition at the intestine and may also be less responsive to P-gp mediated chemosensitization by the named herbs.

## 6. Conclusion

When African herbals that regulate either CYP or P-gp activity were coadministered with conventional drugs they could either synergize or antagonize their activity in addition to exerting their own intrinsic toxicity. As a result, therapeutic drug monitoring systems are needed to examine the extent and severity of such interactions. Nevertheless, beneficial interactions can be medically exploited. For example, pervilleine and tuckeyanols can reverse MDR in tumours. Similarly, when coadministered with* H. sabdariffa* and* T. foenum-graecum,* HCTZ and glibenclamide, respectively, are potentiated and can be administered at lower doses thus reducing their inherent toxicities. Subsequently, compliance too may be enhanced [92].

Our work relied heavily on findings from in vitro studies which tend to have a low predictive value because literature was scarce. Therefore, more clinical research effort needs to be expended in this field in order to generate accurate and reliable data so that health practitioners can be equipped with the necessary skills to anticipate and manage HDIs accordingly and advice patients on traditional medicine use.

Another challenge in Africa emanates from the weak policy and regulatory frameworks regarding traditional medicine [213]. This too can be addressed in part by furthering research that can assist governments to design interventions that adequately regulate traditional medicine practice and possibly integrate it into mainstream healthcare systems.

## Figures and Tables

**Table 1:** Common African herbs that modulate CYP and P-gp activity.

Botanical name	Common name	Bioactive compound	Indication	Target	Probe drug	Effect	Study	References
*Acacia nilotica*	Gum Arabic	Acanilol A, acanilol B, kaempferol	Diabetes	P-gp	Cyclosporin A	Synergism	In vitro	[57–59]

*Aframomum melegueta* (Roscoe) K. Schum	Alligator pepper	Humulene, saryophyllene	Diabetes	CYP3A4, CYP3A5, CYP3A7	Ketoconazole	Synergism	In vitro	[60, 61]

*Bixa orellana*	Lipstick tree	Bixin	Diabetes	CYP1A1/2, CYP2B1/2, CYP3A	Pentoxyresorufin, Benzyloxyresorufin	Antagonism	In vitro	[62, 63]

*Capsicum annuum*	Red chilli pepper	Capsaicin	Hypertension	P-gp	Daunorubicin, Rhodamine-123	Synergism	In vitro	[64]

*Carica papaya*	Pawpaw	Carpain, alkaloids, terpenes, flavanols	Diabetes	P-gp,	Digoxin, amiodarone	Synergism	In vivo, in vitro	[65–67]

*Catha edulis*	Khat	Cathinones	Diabetes	CYP2D6	Dextromethorphan	Synergism	In vitro/in vivo (human)	[68, 69]

*Centella asiatica*	Centella	Triterpenes, asiatic acid	Hypertension	CYP2C9, CYP3A4	Tolbutamide	Synergism	In vitro	[70, 71]

*Citrus aurantifolia* (Christm.) Swingle	Lime	Imperatorin, kaempferol, myricetin, *β*- sitosterol	Diabetes	CYP 2B6	Bupropion	Synergism	In vitro	[72, 73]

*Citrus aurantium *L.	Sour orange	Methoxyflavones	Diabetes	Intestinal CYP3A4, P-gp	Felodipine Vinblastine	Synergism	In vivo (human)	[74, 75]
		Auraptene, nobiletin	Cancer	P-gp	Daunorubicin	Synergism	In vitro	[76]

*Corchorus olitorius*	Long fruited jute	3,5-Dicaffeoylquinic acid	Diabetes	CYP3A4	Ketoconazole	Synergism	In vitro	[60, 77]

*Curcuma longa*	Turmeric	Curcumin	Diabetes	CYP3A4, CYP2C8, CYP2C9, CYP1A2, CYP2A6, CYP2D6, CYP2B6	Pioglitazone, caffeine, daunorubicin	Synergism/antagonism on CYP2A6	In vitro, in vivo (human)	[78–81]

*Ipomea batatas*	Sweet potato	6-*O*-caffeoylsophorose, 4-ipomeanol acylated cyanidin, peonidin	Diabetes	P-gp	Rhodamine-123	Synergism	In vitro	[82]

*Jatropha curcas *L.	Purging nut	Coumarins, Jatrophalactam, Jatrogrossidione derivatives	Diabetes	CYP3A4, CYP3A7	Ketoconazole	Synergism	In vitro	[60, 83]

*Morinda lucida *Benth.	Brimstone tree	Anthraquinones	Diabetes	P-gp	Digoxin	Synergism	In vitro	[65, 84]

*Murraya koenigii *(L.)Spreng.	Sweet neem	Girinimbine, mahanimbilol, koenimbine, Xanthotoxin	Diabetes	CYP1A2, CYP2C9, CYP2D6, CYP3A4	Ketoconazole, quinidine, sulfaphenazole, *α*-naphthoflavone	Synergism	In vitro	[85–87]

*Persea americana *Mill.	Avocado pear	Caryophyllene oxide, *α*- and *β*-pinene, caryophyllene	Diabetes	CYP 3A4, CYP3A5, CYP3A7	Ketoconazole	Synergism	In vitro	[60, 88]

*Sesamum indicum*	Sesame	Sesamin	Dietary nutrient	P-gp (MDR1)	Daunorubicin	Synergism	In vitro	[89]

*Vernonia amygdalina*	Bitter leaf	Sesquiterpene lactones, steroidal glycosides	Diabetes	P-gp	Digoxin	Synergism	In vitro	[65, 90]
